# Automated segmentation of the corneal endothelium in a large set of ‘real-world’ specular microscopy images using the U-Net architecture

**DOI:** 10.1038/s41598-019-41034-2

**Published:** 2019-03-18

**Authors:** Moritz C. Daniel, Lisa Atzrodt, Felicitas Bucher, Katrin Wacker, Stefan Böhringer, Thomas Reinhard, Daniel Böhringer

**Affiliations:** 1 Eye Center, Medical Center, University of Freiburg, Freiburg, Germany; 2 Department of Biomedical Data Sciences, Leiden University Medical Center, Leiden, The Netherlands

## Abstract

Monitoring the density of corneal endothelial cells (CEC) is essential in the management of corneal diseases. Its manual calculation is time consuming and prone to errors. U-Net, a neural network for biomedical image segmentation, has shown promising results in the automated segmentation of images of healthy corneas and good quality. The purpose of this study was to assess its performance in “real-world” CEC images (variable quality, different ophthalmologic diseases). The outcome measures were: precision and recall of the extraction of CEC, correctness of CEC density estimation, detection of ungradable images. A classical approach based on grayscale morphology and water shedding was pursued for comparison. There was good agreement between the automated image analysis and the manual annotation from the U-Net. R-square from Pearson’s correlation was 0.96. Recall of CEC averaged 0.34 and precision 0.84. The U-Net correctly predicted the CEC density in a large set of images of healthy and diseased corneas, including images of poor quality. It robustly ignored image regions with poor visibility of CEC. The classical approach, however, did not provide acceptable results. R-square from Pearson’s correlation with the ground truth was as low as 0.35.

## Introduction

The corneal endothelium comprises confluent polygonal corneal endothelial cells (CECs). These form a monolayer that completely lines the inner surface of the cornea. CECs are vital for corneal transparency since they regulate the water content of the stromal layer.

At birth, humans possess a large functional reserve of 3000 to 5000 CECs per square millimeter. CECs are postmitotic and do not regenerate in case of damage or idiopathic age-related loss. Instead, neighbouring CECs elongate and migrate to fill the gaps^1^. Excessive loss of CECs below a number of approximately 300 to 500 cells per square millimeter results in painful epithelial damage and potentially blinding corneal edema. This condition ultimately requires endothelial transplantation^2,3^.

Before this happens, chronic CEC loss is commonly asymptomatic. Direct monitoring of CECs is therefore essential in safety assessments and disease management.

CEC density is the most important parameter for this purpose. Calculating the CEC density requires that the CECs are individually located, e.g. by means of ‘dotting’ the centroids with a computer mouse^4^. Sampling error considerations call for dotting as many CECs as possible^5^. Automated CEC segmentation has been pursued for decades in order to facilitate this task. To date, the majority of methods rely on marker-driven watershed segmentation^6–9^. This method, however, is prone to under- as well as to over-segmentation, i.e. in areas with poor image quality^10^.

Several methods using predefined image features have been proposed to tackle this difficult problem^11,12^. Poletti *et al*. demonstrated that using a filter-bank of three features achieves reliable segmentation when combined with a supervised classification step^12^. The small number of 20 validation images in this study makes it difficult to judge in how far results generalize to a heterogeneous corpus. The step of defining “hand-crafted” features is arguably likely to induce over- and under-segmentation when a model is applied to a broader spectrum of real-world CEC images than seen in the training phase.

Scarpa *et al*. proposed a completely different solution. They iteratively fitted hexagonal meshes to the pixel intensities with the help of genetic optimisation. This creative approach showed promising results for cell border detection in a pilot study with 30 images^13^. However, this method may be disturbed by guttae and/or descemet’s folds within the central parts of the image. This has not been tested so far to the best of our knowledge.

Neural networks gave rise to alternative segmentation approaches without the need for pre-specified features^10,14,15^. The convolutional U-Net architecture comprises a neural network that has been developed especially for biomedical image segmentation. When trained on rather a limited number of CEC images this has already shown very promising results^16^. However, U-Net has not been challenged with a large number of ‘real-world’ images from diseased corneas, images with poor quality or images without clearly visible CECs.

We herein present a large corpus of non-contact endothelial specular microscopy images that have been recorded in the cornea service of our highly specialized tertiary referral center. This corpus deliberately comprises some ungradable images in order to assess the performances of the algorithm in fully automated settings. All cells on the images have been dotted carefully by one of three corneal experts. We used a subset of the images to train the U-Net and the remainder for assessing the U-Net performance. In particular, we evaluate precision and recall of the extracted CECs, correctness of the CEC density estimation, and how ungradable images are handled. We also evaluate the same images with the prototypical watershed-driven CEC segmentation method of Vincent for comparison^6^.

## Methods

### Image corpus

We screened the specular microscopy database of currently ~16,000 images from our corneal consultation service for suitable images. We aimed for covering the full spectrum of healthy corneas, corneas with endothelial diseases, and images from corneal grafts at different follow up intervals after keratoplasty. We included images of variable quality, including images with poor illumination as well as images without any visible endothelial cells (e.g. due to corneal edema). Images with guttae were not excluded. We picked only one image from each patient. We used Topcon SP-3000 specular microscopes exclusively. The images had been directly digitized with commercial video grabbers attached to the analog (‘high frequency’) video-printer port of the microscopes. The digitized video prints were uniformly cropped to the rectangular area of the CEC image. All images were 260 pixels wide and 476 pixels high. This step anonymized the data.

The sample size was limited to 385 images (from 385 patients) in order to limit time expenditure required for manual dotting. A team of three experienced physicians manually dotted all CECs. Each image was dotted by one expert. The sole instruction was to click on the visual center (centroid) of every unambiguous cell. The resulting CEC centroid coordinates were stored in a relational database. Images with multiple guttae were flagged.

### Application of the U-Net

The U-Net was originally developed by Ronneberger *et al*.^17^. This method seems to perform very well in CEC segmentation as recently demonstrated with only 15 CEC images used for training^16^. We chose the implementation of Akeret *et al*.^18^ that can be used with a freely available software stack (downloaded from https://github.com/jakeret/tf_unet on June/7/2018).

We randomly selected 158 training images from our corpus which had not been classified as ungradable by the corneal experts to train the U-Net. In order to make the analysis robust against technical variation in image sources, a limited number pre-processing steps were performed. We started with gaussian blurring (radius 1 pixel and standard deviation of 2 pixels) followed by a grayscale morphology ‘Top-Hat’ operation. As a last step, we performed histogram equalisation. We generated the binary label-maps by means of water shedding constrained by the markers from manual dotting. Image processing was done using the ‘EBImage’ package from ‘bioconductor’^19^. In a next step, we split all images into patches of 78 by 78 pixels using the software package ‘ImageMagick’. Each sub image was replicated by means of mirroring horizontally as well as vertically and at both axes. All operations were identically performed on the label images. This resulted in a total of 11,376 image patches for training.

We configured the U-Net to have 3 layers,26 root features, one color channel (grayscale) and two segmentation classes (cell border vs. no cell border). Otherwise the standard U-Net architecture was used (3 × 3 convolutions, 2 × 2 max-pooling) and more details are given elsewhere^17^. We used 200 training iterations with 150 epochs. We opted for the ‘Adam’ optimizer. Training the U-Net took less than one hour on a Mac Pro (Model of 2013, Apple Inc.) using Tensorflow (Version 1.8, Google Inc.). Training time is not a limitation, because the network needs to be trained only once.

**Figure 1:**
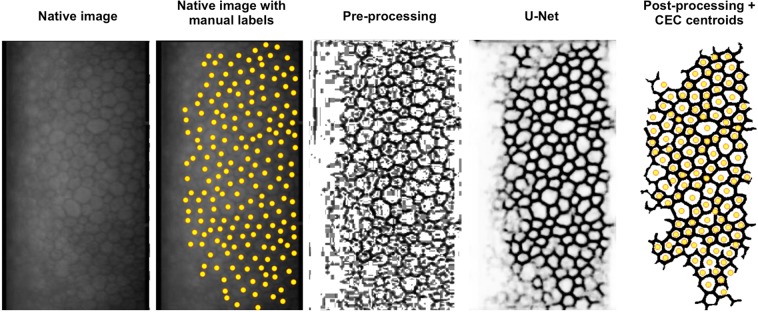
Sequence of image processing with the U-Net. Reference image and reference image with manually labeled centroids on the left. Note how the areas of poor visibility are translated into low border probability (=bright) and how our post processing eventually eliminates these ungradable areas by eliminating the disjointed small blobs.

After training, the neuronal network could transform arbitrary grayscale images into grayscale probability maps with respect to cell borders (Fig. 1). This process took only one second per image without hardware acceleration. The probability maps were thresholded at a probability of 60%. Thereafter, noise and areas with ambiguous cell borders were eliminated by means of removing all objects comprising less than 1000 pixels. This ensured that only the central area with well defined cell borders remained for CEC centroid extraction. Centroids were extracted via local maxima in the distance map of the final image.

### Application of Vincent’s method

**Figure 2:**
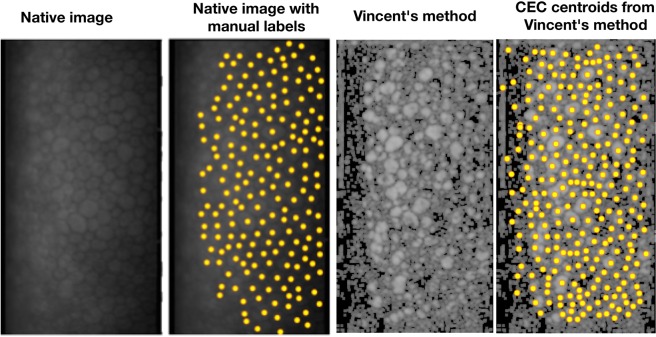
Sequence of image processing with Vincent’s method. Reference image and reference image with manually labeled centroids on the left are the same as in (**a**). Note the high degree of overdetection in areas of poor image quality.

Vincent was the first to develop a robust method of marker-driven watershed segmentation for CEC images^6^. He proposed the following image processing sequence: in a first step, noise is filtered out with an alternate sequence filter (morphological opening followed by morphological closing with structuring elements increasing in size). On this filtered image the morphological ‘hdome’ operation is applied (Fig. 2). The resulting image is subtracted from the original filtered image. The regional maxima in this difference image (‘h-minima’ image) are used to constrain the water shedding of the original image.

### Validation

We evaluated the segmentation performance by metrics based on centroid coordinates because the ground truth was available only in terms of centroid coordinates. We consider this a reasonable approach as all clinically relevant analyses can also be performed on the basis of the CEC centroids alone^20^. Correct centroid detection was defined as coincidence of the extracted centroid coordinates and the manually annotated (‘ground truth’) coordinates. Some degree of tolerance is warranted, because the manual dotting is subject to rater error and the automatic centroid definition by means of the distance map does not necessarily correspond to the exact centroid of the cell shape. We classified cells as accurately detected as long as the centroid was within a radius of 8 pixels around the reference coordinate.

Recall (sensitivity) of CECs can be defined as the number of correctly identified CECs divided by the number of CECs in manual annotation. Precision (positive predictive value) on the other hand can be defined as number of correctly identified CECs divided by the total number of CECs identified by the segmentation method.

However, the most significant benchmark is correctness of the CEC density prediction. We calculated the CEC density from the inverse of the median CEC area for this purpose. The CEC areas in turn had been deduced from the CEC centroids by means of Voronoi tessellation, excluding the border centroids. The parameters pleomorphism and polymegethism are rarely used in routine practice because these parameters cannot be reliably determined in images with guttae or descemet’s folds^21^. CEC density, by contrast, can almost always be robustly extracted from CEC images of sufficient quality, even in the presence of guttae^22^.

We used the R plattform (version 3.4.3) for these computations^23^. The final performance metric is detection of ungradable images. Failure to detect ungradable images would lead to unreliable data in an automated setting. Ungradable images from the corpus are defined by the absence of manual dotting. U-Net output was defined to be ungradable when no centroids had been extracted.

### Ethical considerations

In accordance with the ethics committee of the Albert Ludwigs University, Freiburg we did not obtain informed consent from the patients because we worked on anonymized data that had been extracted retrospectively from the patient records. This study was approved by the ethics committee of the Albert Ludwigs University, Freiburg (10010/18). All methods were performed in accordance with the Declaration of Helsinki.

## Results

### Image corpus

**Figure 3:**
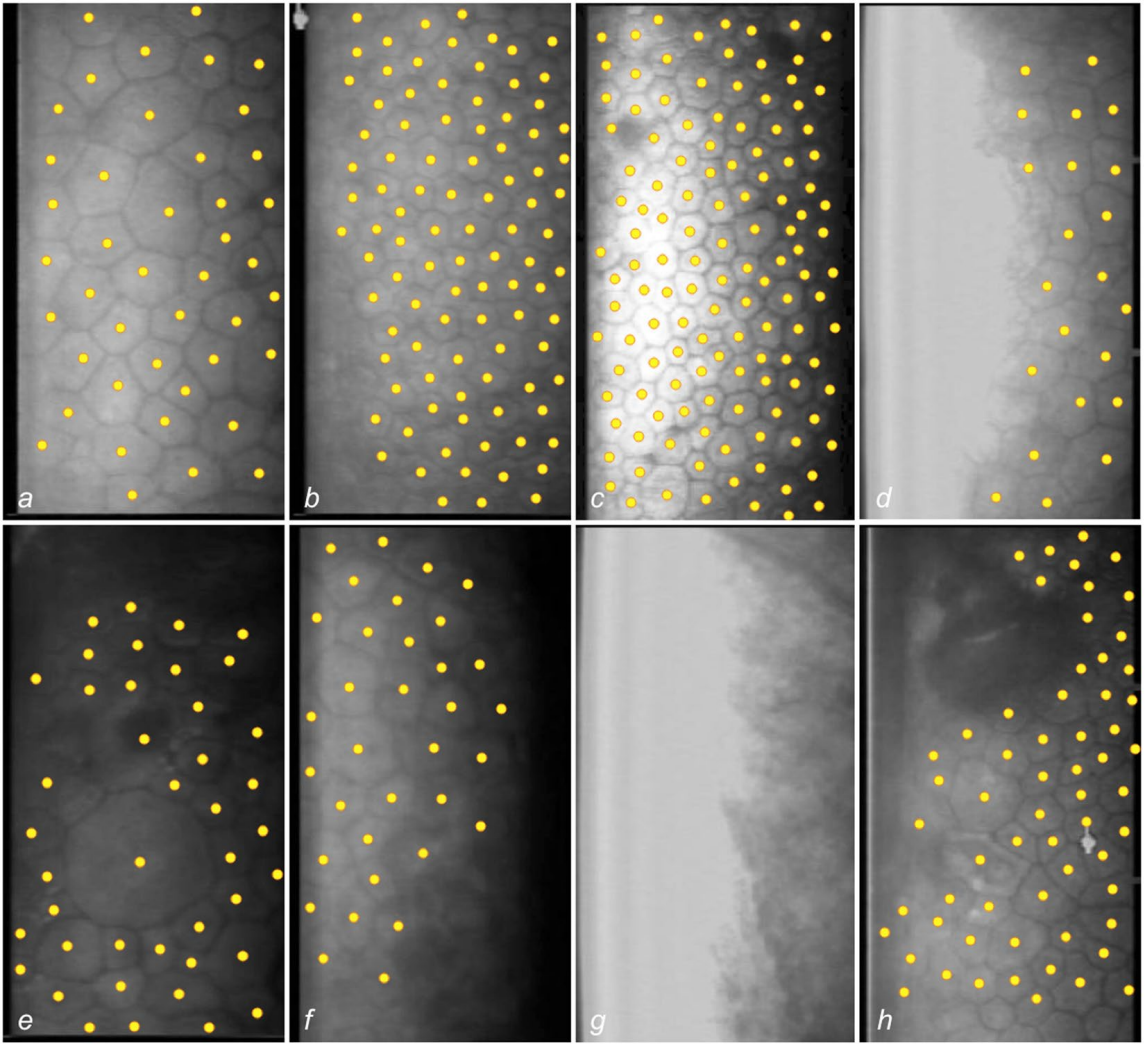
Non-contact specular microscopy images of the corneal endothelium representing the spectrum of images. The cell centroids were marked manually by three corneal experts. (**a**) Uniformly exposed image of well demarcated cells, moderate cell size variability (polymegathism); (**b**) tessellation of cells relatively small in size; *c)* overexposed group of cells on the left (cell borders are visible); (**d**) cells on the left are not visible due to overexposure; (**e**) large cells in the center of the image surrounded by CECs variable in the size and shape (pleomorphism); (**f**) blurred cell margins in the right/bottom right section of the image, due to underexposure; (**g**) endothelial cells not visible due to low image quality; (**h**) large gutta in the top half of the image, pleomorphism and polymegathism.

We included a total 385 reference images representing the full spectrum from healthy to diseased corneas in our corpus. The median number of cells dotted per image was 90 (interquartile range (IQR): 38 to 172). The endothelial cell density averaged 1607 cells per square millimeter (IQR: 871 to 2296). 36 images had multiple guttae. 92 of the images had a cell density of less than 1000 cells per square millimeter. These images originate from post-keratoplasty eyes that suffer from chronic endothelial cell loss. Most ungradable images originate from eyes with end-stage corneal diseases with stromal opacities and/or stromal edema. 61 images were marked ungradable because no endothelial cells were visible. Figure 3 depicts some examples of all categories.

### Performance of the U-Net

The validation set comprised 227 images. The number of cells identified on each image averaged 46 (maximum: 364; IQR: 0 to 74). The recall averaged 0.34 (minimum: 0; maximum: 0.73; IQR: 0.06 to 0.53). The low recall resulted from the elimination of all objects comprising less than 1000 pixels from the binarized U-Net probability map. This step was performed in order to maintain only the central and contiguous cell mosaic with all poor quality image regions removed.

The average precision turned out to be 0.84 (minimum: 0.17; IQR: 0.8 to 0.94). That means that the majority of CECs identified by the U-Net corresponded to the CECs dotted by the corneal experts.

**Figure 4:**
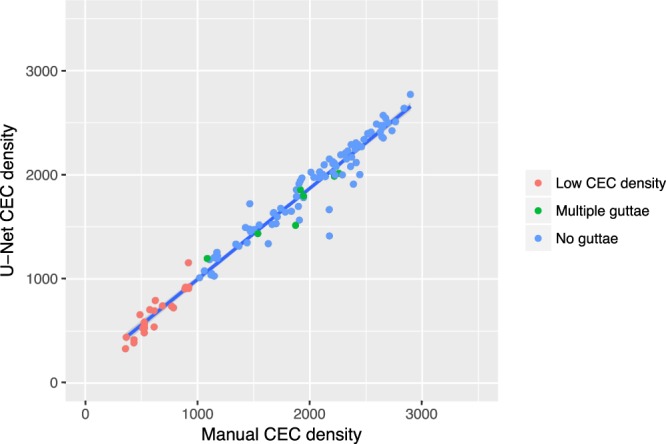
Correlation between manual CEC density and U-Net CEC. R-square is 0.96. Ninety two percent of images agree within a limit of +/−250 cells per square millimeter.

The correlation between manual CEC density and U-Net CEC density revealed good agreement (Fig. 4). This also proved to be valid for images with multiple guttae (green dots) as well as for low CEC density post keratoplasty eyes (red dots).

R-square from Pearson’s correlation is 0.96. Ninety two percent of images agreed within a limit of +/−250 cells per square millimeter, 99% agreed within a corridor of +/−500 cells per square millimeter.

The U-Net classified 92 images as ungradable whereas only 61 had been considered ungradable by the corneal experts. In 26 images, the U-Net failed to detect the CECs that had been dotted by the corneal experts (these were the images of very poor quality showing areas of very low CEC density). In only one ungradable image, the U-Net incorrectly labelled CECs.

### Performance of Vincent’s method

**Figure 5:**
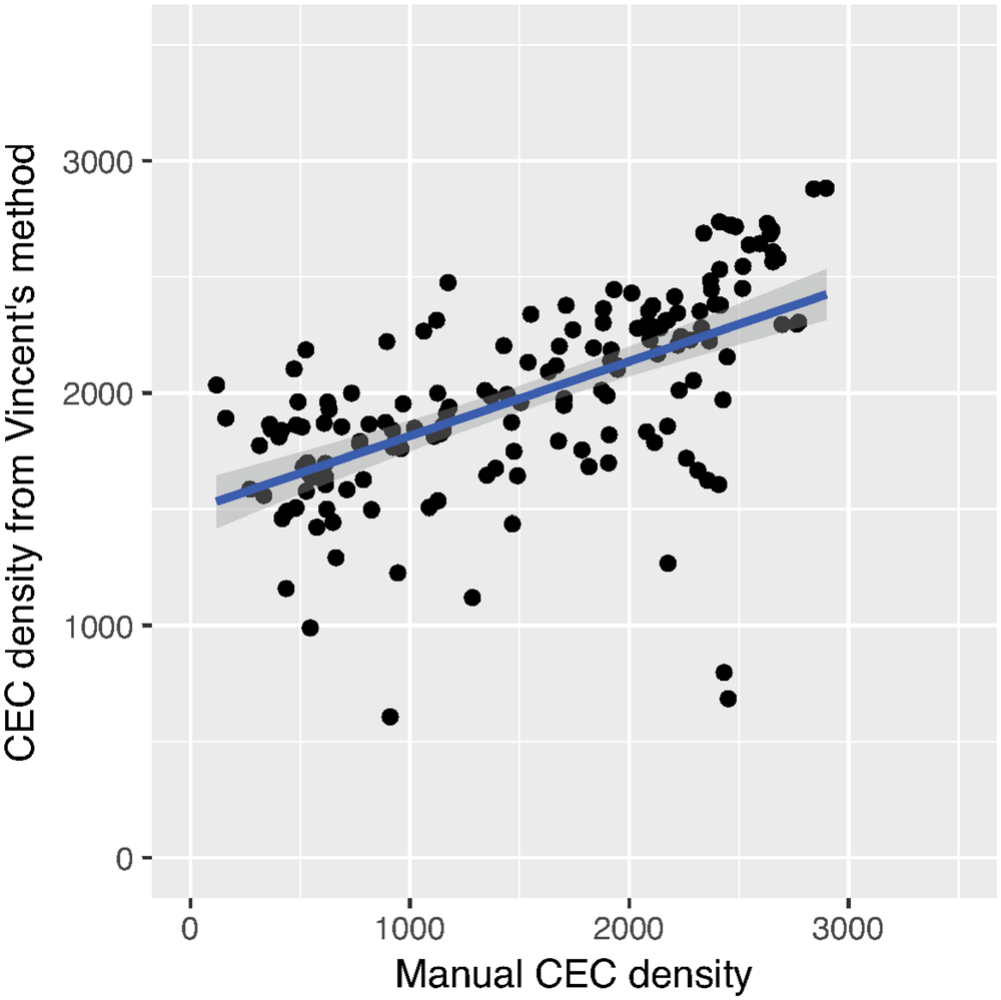
Correlation between manual CEC density and CEC from Vincent’s method. R-square is as low as 0.35. Only 35 percent of images agree within a limit of +/−250 cells per square millimeter. Images with multiple guttae (green dots) and low endothelial cell density (red dots) are color coded to show that the U-Net can handle these conditions sufficiently well.

The number of cells identified on each image averaged 95 (maximum: 284; IQR: 36 to 146). The correlation between manual CEC density and U-Net CEC density was unacceptably low for clinical and research purposes. R-square from Pearson’s correlation is only 0.35 (Fig. 5). Only 35% of all analyses agreed within a limit of +/−250 cells per square millimeter.

## Discussion

The purpose of our investigation was to assess the U-Net performance in a large set of real-world specular microscopy CEC images and to compare this promising method with a classical method based on grayscale morphology and water shedding. Our first step was to compile an annotated image corpus that covers the full spectrum of healthy and diseased corneas and corneal grafts, including images without any visible CECs.

Images from non-contact endothelial microscopy are particularly well suited for benchmarking CEC segmentation. This non-invasive method is nowadays well established in the clinical routine, whereas the contact methods are used less frequently, mostly in scientific projects. Images from non-contact microscopy vary vastly in imaging quality because tear film irregularities can severely interfere with image contrast and exposure, which is not the case in the more laborious and invasive methods of contact microscopy. Our corpus intentionally covers the full spectrum of corneal diseases seen in a cornea specialist hospital because corneal opacities, guttae and stromal edema all interfere with image quality and visibility of the CEC borders^4^.

We opted for annotating the corpus only with the centroids coordinates of the CECs and we demonstrate that it is possible to train the U-Net on this basis. Hand-drawn label-map images on the other hand would have been much more time consuming to produce. This simplification enabled us to include a total of 385 annotated images into the corpus. Additionally, dotting is most likely associated with a lower rater error when allowing for a neighborhood in the cell for the centroid definition, although we did not confirm this by re-labeling experiments.

The centroids are also excellent for benchmarking. Recall and precision have already been proposed by Selig *et al*.^8^ These metrics are sensitive to both over- and under-segmentation. Moreover, these simple concepts are vividly understandable by clinicians and clinical scientists, as is the correctness of CEC density estimations. It is currently not clear whether the more complex distance metrics at the pixel-level have any additional clinical benefit.

The low recall contrasts with a high correlation of the CEC density estimates with the ground truth. The low recall resulted from the elimination of all objects comprising less than 1000 pixels from the binarized U-Net probability map. The recall could possibly be improved by means of introducing a second learning objective into the neural net: whether a given cell border lies on the border region. This so-called multitask learning has been successfully employed in facial application where forcing the network to learn facial pose improves internal representations and consequently landmarking performance, the main goal of that network^24^.

The tight correlation of CEC density estimation with the manual input indicates that the U-Net is suitable for fully automated settings. Interestingly, this seems to hold for images with multiple guttae and images from eyes with low cell density from chronic endothelial cell loss (see colored dots in Fig. 4).

This is not the case with the classical approach based on grayscale morphology and water shedding. Vincent’s method e.g. is prone to overdetection of cells in areas with poor image quality. The currently common solution is to manually define the ‘region of interest’ comprising a contiguous region of good CEC visibility. This manual interaction is not required by the U-Net because regions with poor visibility of CEC borders can be automatically excluded from analysis. As a consequence, the U-Net is to the best of our knowledge the first method that can detect (and ignore) ungradable images at high sensitivity. This not only lowers the human workload but also increases objectivity and reproducibility.

Although the U-Net is not directly useful for classification purposes, the extracted cell centroids can be used for this using machine learning. This could be used e.g. to discriminate between normal and pathologic endothelium or for detecting nascent immune reactions following keratoplasty. The automated extraction of a large number of contiguous CEC centroids also paves the way for introducing the concept of CEC mosaic tracking and other methods that require automated CEC dotting into the clinical routine^25–27^.

Last but not least, we are optimistic that our corpus may be of some value to computer vision/segmentation scientists that do not have direct access to a large number of ‘real-world’ specular microscopy images.

## Data Availability

The datasets and images generated during and/or analysed during the current study are available from https://github.com/daboe01/SREP-18-33533B or from the corresponding author on reasonable request.
